# Complexome Profiling—Exploring Mitochondrial Protein Complexes in Health and Disease

**DOI:** 10.3389/fcell.2021.796128

**Published:** 2022-01-12

**Authors:** Alfredo Cabrera-Orefice, Alisa Potter, Felix Evers, Johannes F. Hevler, Sergio Guerrero-Castillo

**Affiliations:** ^1^ Center for Molecular and Biomolecular Informatics, Radboud Institute for Molecular Life Sciences, Radboud University Medical Center, Nijmegen, Netherlands; ^2^ Department of Pediatrics, Radboud Center for Mitochondrial Medicine, Radboud University Medical Center, Nijmegen, Netherlands; ^3^ Department of Medical Microbiology, Radboud Institute for Molecular Life Sciences, Radboud University Medical Center, Nijmegen, Netherlands; ^4^ Biomolecular Mass Spectrometry and Proteomics, University of Utrecht, Utrecht, Netherlands; ^5^ Bijvoet Center for Biomolecular Research, University of Utrecht, Utrecht, Netherlands; ^6^ Utrecht Institute for Pharmaceutical Sciences, University of Utrecht, Utrecht, Netherlands; ^7^ Netherlands Proteomics Center, Utrecht, Netherlands; ^8^ University Children’s Research@Kinder-UKE, University Medical Center Hamburg-Eppendorf, Hamburg, Germany

**Keywords:** complexome profiling, mitochondria, protein complex, protein-protein interaction (PPI), oxidative phosphorylation, disease, proteomics, mass spectrometry

## Abstract

Complexome profiling (CP) is a state-of-the-art approach that combines separation of native proteins by electrophoresis, size exclusion chromatography or density gradient centrifugation with tandem mass spectrometry identification and quantification. Resulting data are computationally clustered to visualize the inventory, abundance and arrangement of multiprotein complexes in a biological sample. Since its formal introduction a decade ago, this method has been mostly applied to explore not only the composition and abundance of mitochondrial oxidative phosphorylation (OXPHOS) complexes in several species but also to identify novel protein interactors involved in their assembly, maintenance and functions. Besides, complexome profiling has been utilized to study the dynamics of OXPHOS complexes, as well as the impact of an increasing number of mutations leading to mitochondrial disorders or rearrangements of the whole mitochondrial complexome. Here, we summarize the major findings obtained by this approach; emphasize its advantages and current limitations; discuss multiple examples on how this tool could be applied to further investigate pathophysiological mechanisms and comment on the latest advances and opportunity areas to keep developing this methodology.

## 1 Introduction

Proteins, the so-called “workhorses” of life, are the tools that make living machines work (Adams, 2008). The vast majority of biological processes are thus structured, mediated and executed by these macromolecules. Even though many single proteins do perform specific functions by their own, most proteins habitually interact with other proteins, DNA, RNA and lipid molecules rather than acting individually for achieving their biological tasks. The resultant protein complexes can form transient or steady interactions, which also correlate with the type of biological processes they are involved in (De Las Rivas and Fontanillo, 2010). For example, housekeeping cell processes are likely performed by a large fraction of steady protein complexes, whereas in signaling, cell migration, membrane trafficking, metabolic response and other highly dynamic processes, involvement of transient complexes is definitely required for rapid responses and adaptation. The entire set of multi-protein complexes in a cell or compartment is referred to as the complexome (Deshaies et al., 2002; Ceulemans et al., 2006; Lasserre et al., 2006).

In situations where impaired interaction of the elements of a protein complex affect its proper formation; e.g., due to genetic mutations, the related cell process(es) could be compromised and result in biological dysfunction. Higher complexity in multicellular organisms is of course accompanied by larger complexomes than those from unicellular species. Therefore, alterations in protein complexes may affect not only multiple cell processes, but also lead to severe pathophysiological issues at the tissue/organ level. An integral elucidation of both complexomes and protein interaction networks; i.e., interactome (Vidal et al., 2011) under different cellular scenarios, becomes crucial to fully comprehend the molecular mechanisms behind cell physiology and disease.

Numerous biochemical, biophysical, structural, genetic, microscopy and mass spectrometry (MS) approaches have been applied to characterize protein complexes. Although these methods have different principles, in most cases, they require experimental interventions, cell/tissue fractionation, protein extraction and/or time-consuming protocols prior to data collection and analyses. Besides, the amount of information obtained is often limited to one or a small set of protein complexes. The recent breakthrough in quantitative high-throughput technologies and bioinformatic tools has substantially increased the efficiency and quality of the large-scale identification of protein interactors (Iacobucci et al., 2021; Low et al., 2021). Accordingly, an outstanding volume of evidence on the composition, 3D structure, interactions and molecular roles of hundreds of protein complexes is now easily accessible through multiple repositories, such as RCSB PDB (Burley et al., 2021), STRING (Szklarczyk et al., 2021), CORUM (Giurgiu et al., 2019), BioGRID (Oughtred et al., 2021), Pfam (Mistry et al., 2021), Complex Portal (Meldal et al., 2021), NCBI (Coordinators, 2016) and UniProt (UniProt, 2021). Yet, a substantial fraction of the protein interactors reported in those databases still lack full validation of their occurrence *in vivo* by using novel and more reliable methods.

A systematic approach known as complexome profiling (CP) has recently been introduced as a straightforward and unbiased way to explore protein complexes (Heide et al., 2012; Wessels et al., 2013). CP combines fractionation of protein complexes under native conditions with digestion and quantitative tandem MS-based identification of the derived peptides. Resultant data are clustered to visualize the inventory, abundance and arrangement of multi-protein complexes in a biological sample. Since its formal introduction a decade ago, CP has been primarily used in mitochondrial research (Kiirika et al., 2013; Senkler et al., 2017; Giese et al., 2021; Guerrero-Castillo et al., 2021b; Wittig and Malacarne, 2021); albeit CP can be applied to any kind of cellular fraction.

## 2 Complexome Profiling

### 2.1 General Overview

**FIGURE 1:**
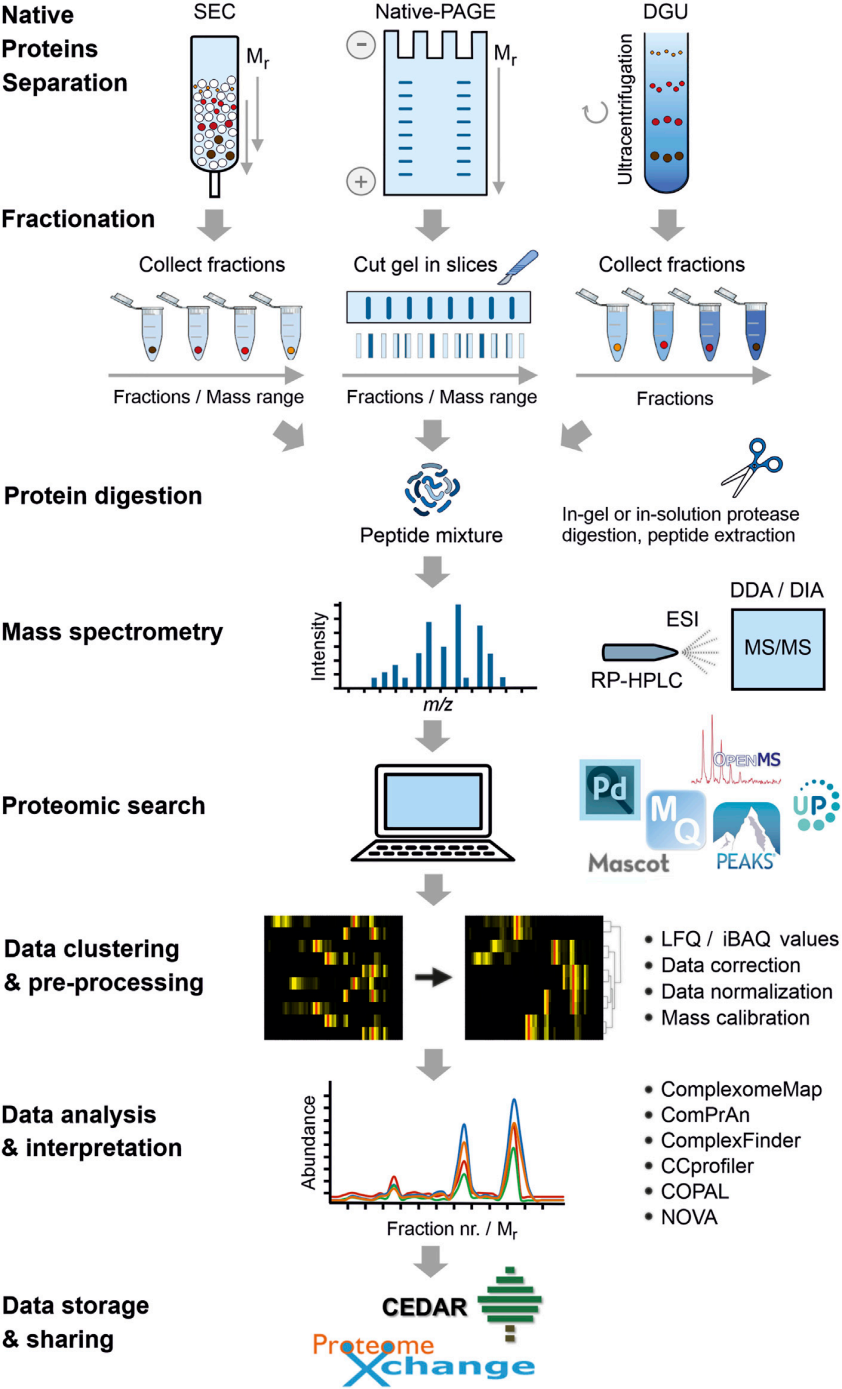
Overall workflow of complexome profiling (CP). After collection, homogenization and fractionation of biological materials, proteins are separated by either native polyacrylamide gel electrophoresis (PAGE), size exclusion chromatography (SEC) or density gradient ultracentrifugation (DGU) for CP studies. The obtained protein-containing fractions are individually digested with specific proteases. Resultant peptides are extracted, cleaned and usually separated by reversed-phase high-performance liquid chromatography (RP-HPLC) followed by tandem mass spectrometry (MS/MS) analysis. MS data can be acquired in data-dependent or data-independent modes, DDA or DIA, respectively. Next, a proteomic search is performed to match obtained MS spectra against proteome databases using a variety of available software. Icons of the most popular tools used for CP studies are shown (see Section 2.1 for details). Protein abundance profiles are further obtained by plotting LFQ/iBAQ values against the number of fractions. The list of identified protein groups is computationally sorted based on similarities of the abundance patterns across fractions; e.g., hierarchical clustering. Prior to analysis, complexome data are pre-processed to account for protein loading/MS sensitivity differences. Data correction and normalization between samples are regularly applied. In SEC- and native-PAGE-based CP, a mass calibration can be implemented for a more meaningful biological dimension. Data can be analyzed by using available bioinformatic tools specifically created for CP. Some of these programs are shown in the figure (see Section 2.4 for details). For reusing and further analysis, large CP datasets can be deposited and publicly shared through PRIDE (Proteome Xchange consortium) or the new dedicated website for CP, CEDAR.

The first requisite to carry out a CP experiment is the collection of biological material; e.g., tissue pieces/biopsies, cultured cells, enriched cell fractions, purified organelles, etc. These materials might also come from previous experimental interventions. Homogenization and cell fractionation methods should aim to keep the native state of protein complexes. For solubilization of membrane proteins, it is recommended to use mild, non-ionic detergents; e.g., Triton X-100, NP-40, dodecyl-maltoside, octyl-glucoside or digitonin (Eubel et al., 2005; Wittig et al., 2006). Next, native protein extracts are separated by a so-called “untargeted” method (Iacobucci et al., 2021), such as native electrophoresis, size-exclusion chromatography or density gradient ultracentrifugation (Figure 1). Regardless of the type of protein separation, CP follows a well-defined protocol that slightly differs among variants. In all cases, protein complexes are separated by size, hence, by shape and molecular mass. Each fraction is further digested with high-purity and specific proteases (e.g., trypsin, chymotrypsin and endoproteinase Lys-C) followed by MS/MS identification of the resulting peptides (Figure 1); i.e., bottom-up proteomics strategy (Zhang et al., 2013).

Previous to MS analysis, peptides are usually separated based on their hydrophobicity by reversed-phase high-performance liquid chromatography (RP-HPLC) followed by electrospray ionization (ESI) to produce gas phase ions (Zhang et al., 2013). The majority of MS data for CP studies has been acquired in data-dependent mode (DDA); i.e., collection of a predefined number of precursor ions for fragmentation during the MS2 cycle is done according to their charge states and abundance (Hu et al., 2016). In conventional DDA modes, the possibility to identify low abundant peptides thus becomes significantly limited. To avoid this issue, acquisition strategies in data-independent mode (DIA) (Krasny and Huang, 2021), such as sequential window acquisition of all theoretical mass spectra (SWATH-MS) (Gillet et al., 2012), in which fragmentation of all precursor ions identified in the MS1 cycle can be analyzed in the MS2 cycle, have recently been introduced to CP-like workflows (Heusel et al., 2019; Bludau et al., 2020; Calvo et al., 2020).

MS spectra are routinely matched against a proteome database of the species of interest by using a variety of software/search engines (Figure 1), such as Mascot (Perkins et al., 1999), openMS (Rost et al., 2016), PEAKS DB (Zhang et al., 2012), Proteome discoverer (Orsburn, 2021), Protein Prospector (Chalkley et al., 2005) and MaxQuant (Tyanova et al., 2016a). In case that the proteome of the studied organism is not yet available or fully annotated, metagenome or transcriptome data are useful to generate a list of proteins and perform the search. Alternatively, peptide sequences might be deduced directly from tandem MS spectra by using database-independent computational approaches; i.e., *de novo* peptide sequencing (Tran et al., 2017).

Protein abundance profiles are further obtained by plotting, for example, label-free quantification (LFQ) (Cox et al., 2014) or intensity-based absolute quantification (iBAQ) (Schwanhausser et al., 2011; Tyanova et al., 2016a) values against the number of fraction. While LFQ intensities are typically utilized for comparing relative amounts of proteins in multiple samples, iBAQ values offer a more stoichiometric impression of the identified protein groups as those are proportional to their molar quantities. iBAQ values are calculated as the sum of all individual peptide intensities of a given protein group divided by its number of theoretical identifiable peptides. It is thus not surprising that the majority of CP studies use iBAQ since it enables a fair comparison between different protein groups in the same and multiple samples. Additional information regarding LFQ and other alternative methods have been reviewed in Fabre et al. (2014) and Wittig and Malacarne (2021). Ultimately, the list of hundreds or even thousands of identified proteins is mainly sorted based on similarities of the abundance patterns across fractions by hierarchical clustering analysis (Figure 1). Proteins that are part of the same complex consistently cluster together since these show co-migration in the same fraction(s) and similar abundance profiles.

### 2.2 Different Complexome Profiling Setups, One Common Goal

As a ground-breaking “-omics” method, CP has been rapidly developing and spreading among the scientific community. It is thus not striking that in less than a decade, multiple strategies have complemented or even improved its scope. At the same time, several limitations of CP have been circumvented gradually by including novel MS-based strategies, specific adaptions in sample processing and new tools for data analysis. All variants of CP do share a common goal nonetheless: to unravel the composition of protein complexes, as well as their abundance, stabilities, apparent molecular masses and stoichiometries.

The most used CP workflow, currently referred to as “classic CP” (Wittig and Malacarne, 2021), uses Blue Native-polyacrylamide gel electrophoresis (BN-PAGE) followed by MS identification. The main advantages of BN-PAGE are the relatively low amounts of biological material required and high-resolution separation of native proteins. BN-PAGE conditions keep proteins in similar native states to those occurring *in vivo* (Wittig et al., 2006). However, if the presence of Coomassie blue dye affects the stability of one or more protein complexes of interest, milder dye-free versions of native-PAGE can easily be used instead; e.g., high-resolution clear native-PAGE (hrCN-PAGE) (Wittig et al., 2007; Ladig et al., 2011). Native-PAGE is suitable for separation of proteins between ∼0.02–10 MDa. If the study requires interrogation of bigger protein complexes (>10 MDa), large pore BN-PAGE (LP-BN-PAGE) can be applied instead (Strecker et al., 2010; Heide et al., 2012).

After electrophoresis, entire gel strips are fixed and often cut into 32–70 slices followed by LC-MS/MS identification (Heide et al., 2012; Senkler et al., 2017; Vidoni et al., 2017; Giese et al., 2021). For CP analysis, slice numbers can be transformed into apparent molecular mass values by carrying out a calibration using standard proteins as exemplified in Wittig et al. (2010) and Heide et al. (2012). Molecular mass accuracy is optimized by using different sets of standard protein complexes, one for water-soluble proteins and the other for membrane proteins. On the whole, the more fractions collected the better resolution of the resultant profiles will be. In 2016, Müller and co-workers developed cryo-slicing Blue Native-Mass Spectrometry (csBN-MS), which helped increase the resolution, accuracy and quantification of the complexome profiles by slicing the BN-lanes in 230 pieces as well as optimizing MS analysis and protein identification (Muller et al., 2016). The resolution of complexome profiles obtained from ≤30 slices would not be suitable to unambiguously assign protein interactions.

Besides native-PAGE, size exclusion chromatography (SEC) and density gradient ultracentrifugation (DGU) have been implemented in different CP setups. Using SEC, native proteins are separated by filtration through a gel matrix (resin), which consists of spherical porous beads of different sizes depending on the desired range of molecular masses (Burgess, 2018). Elution of heavier proteins is faster than the lighter ones by this method. Apparent molecular masses of eluted fractions can be determined with proper standard mixes; although the mass calibration is more accurate for globular proteins (Hong et al., 2012; Korepanova and Matayoshi, 2012). SEC resins are suitable for separating proteins in a wide range of molecular masses; e.g., 1–700 kDa (Sephadex™, Superdex™); 5–5,000 kDa (Superose™) and 0.01–40 MDa (Sepharose™). The number of fractions that can be collected after SEC is comparable to those aforementioned. For example, in the recently introduced SEC-SWATH-MS approach (Heusel et al., 2019), 81 fractions were collected, subjected to MS identification and examined by complex-centric proteome analysis. This CP setup led to higher sensitivity and accuracy in protein quantification, less noise, and validation of protein interactions. SEC has been particularly useful in CP studies characterizing large protein complexes, such as nuclear components (Connelly et al., 2018). However, key limitations of SEC-involving CP setups would be the larger amounts of biological material required, loss of protein interactions by dilution, formation of self-oligomers and inadequate identification of membrane and low abundant protein complexes (Burgess, 2018; Heusel et al., 2019; Iacobucci et al., 2021).

On the other hand, DGU uses solutions of different densities made of glycerol, sucrose, cesium chloride, iodixanol or Ficoll® through which protein complexes are separated based on their sedimentation rates, where heavier complexes sediment faster. DGU is an excellent technique for separating large protein complexes, ribosomes, membrane vesicles and subcellular organelles. DGU is also suitable to separate cleared cell lysates as well as immunoprecipitation (IP)-captured protein complexes (Lee and Skalnik, 2013; Caudron-Herger et al., 2019). After separation, most of resolved protein complexes remain in near-native states. In a recent study, a reliable DGU-based CP variant, referred to as quantitative density gradient analysis by mass spectrometry (qDGMS) has been developed to study the human mitoribosome (Palenikova et al., 2021a). qDGMS combines stable isotope labeling by amino acids in cell culture (SILAC) and DGU followed by fractionation and LC-MS/MS analysis. SILAC is a MS-based technique that quantifies the differences in protein abundance/expression among biological samples (Ong et al., 2002). In conventional SILAC, two different cell lines/strains are cultured using media supplemented with either “heavy” or “light” essential aminoacids that are labeled with non-radioactive isotopes (e.g., ^13^C, ^2^H, ^18^O, ^15^N) or unlabeled, respectively (Geiger et al., 2011). The specific labeled aminoacids are hence incorporated into all cell proteins during cell growth. Proteins of cell lysates/fractions obtained from the two samples are mixed (1:1), digested and analyzed together by LC-MS/MS. The isotope-labeled peptides appear in MS spectra as pairs with identic chemical composition but different masses. Ratios of intensities for the many identified peptide pairs thus denote the respective changes in protein abundance between samples. Incorporation of SILAC not only in qDGMS but also in classic CP allows duplexing, MS time-saving and, most important, higher accuracy of quantification in proteomic analysis of two experimental conditions (Palenikova et al., 2021a; Palenikova et al., 2021b).

A major challenge of DGU relies on retrieving of fractions without manual disturbance of the resolved layers. To account for this issue, several strategies and devices have been developed, including commercially available automatic fractionators and freezing the gradient after centrifugation followed by cryo-slicing to fractionate samples consistently (Yu et al., 2016). Location of resolved proteins by DGU is considerably more spread when compared to native-PAGE or SEC, which means that increasing the number of fractions does not necessarily lead to higher resolution. Furthermore, sedimentation rates do not only depend on molecular masses of protein particles but also on shape and densities from both the particles and fluid used for making the gradient (Cole et al., 2008). For these reasons, co-migration of identified proteins by this CP setup does not immediately represent actual associations rather than merely similar sedimentation rates.

### 2.3 Other Mass Spectrometry-Based Approaches Related to Complexome Profiling

Additional untargeted MS-based assays have been described in the literature, which share the same principles of a CP experiment. The group of Matthias Mann developed protein correlation profiling (PCP) by combining DGU and quantitative MS to identify and validate several components of the human centrosome (Andersen et al., 2003). A few years later, Harner and co-workers applied PCP to discover the mitochondrial contact site (MICOS) complex (Harner et al., 2011). The further inclusion of hierarchical clustering and bioinformatic analysis of PCP data improved substantially this method leading to a broader characterization of both organelle and cell complexomes (Foster et al., 2006; Havugimana et al., 2012). PCP has also been related to co-fractionation coupled to MS (CoFrac-MS) experiments (Bludau, 2021). In CoFrac-MS, ion-exchange chromatography (IEX) or SEC have however been used for a higher resolution separation of protein complexes by their charges or hydrodynamic radii, respectively (Bludau and Aebersold, 2020). The combination of PCP/CoFrac-MS with other techniques, such as SILAC or stable isotope labelling in mammals (SILAM) have been proven valuable in proteome-scale interactome studies across cells (Kristensen et al., 2012) and tissues (Skinnider et al., 2021).

CP-like approaches using BN-PAGE followed by MS analysis have also been applied in different studies. A systematic proteomic analysis of series of 15 protein bands excised from individual BN-gel lanes allowed the identification of subunits of the mitochondrial respiratory complexes and was helpful to correctly re-assign NDUFA4, previously misidentified as a complex I subunit, as a *bona fide* component of the cytochrome *c* oxidase (Balsa et al., 2012). Likewise, Cogliati and co-workers separated mitochondrial proteins from mice by BN-PAGE and cut the strips in 26 slices before MS identification (Cogliati et al., 2016a) using a data-independent scanning (DiS) method (Guaras et al., 2016). The latter workflow has recently been standardized and referred to as Blue-DiS (Calvo et al., 2020).

### 2.4 Complexome Profiling Data Analysis and Visualization

Huge output files are obtained in CP studies after protein searches, which usually contain all data necessary for further analysis, including identified protein groups, unique peptides, sequence coverage, MS/MS counts, scores, LFQ/iBAQ values from each fraction, etc. The most common visualizations of a complexome profile are heatmaps accompanied by line charts plotting the LFQ/iBAQ values throughout the fractions (Figure 1). Complexome profiles of a short set of proteins can be manually generated and analyzed. Yet, the large volume of protein identifications in CP datasets makes full manual inspection impractical. In the last years, several tools have been specifically designed for automated processing and exploration of CP datasets.

ComplexomeMap has been developed as an online public platform for mining the mitochondrial complexome of plants *Arabidopsis thaliana* and *Viscum album* (Senkler et al., 2017). NOVA is a user-friendly software to perform cluster analysis, mass calibration, normalization, visual inspection, links to protein databases and comparison of experimental conditions (Giese et al., 2015). The software COPAL has proven helpful for analyzing multiple CP datasets, aligning experimental replicates and detecting significantly affected protein complexes (Van Strien et al., 2019). It also generates files that can be directly used for gene set enrichment analysis. ComPrAn, a Shiny R app, has been developed for analyzing qDGMS data; this tool enables analysis of peptide-level data, normalization, clustering, visualization options and a graphical user interface (Palenikova et al., 2021a). In addition, ComPrAn is particularly useful to analyze proteomic data obtained from SILAC-treated samples. ComplexFinder, a Python-based software suit, has recently been released to analyze fractionation of native protein complexes, particularly from BN-PAGE- or SEC-based CP experiments (Nolte and Langer, 2021). This tool allows machine learning-based prediction of potential protein-protein interactions (PPIs) and high flexibility in CP data analysis. ComplexFinder also provides improved peak-centric quantification and kinetic modelling, protein connectivity networks and compatibility with different quantification strategies. CCprofiler is a robust R package for analyzing co-fractionation MS datasets, which has been originally designed for SEC-SWATH-MS (Heusel et al., 2019). CCprofiler and its web interface, SECexplorer, offer multiple functions, such as quality control and filtering for less erroneous assignment of protein interactors, usage of curated reference datasets, protein quantification, protein- and complex-centric data analysis and visualization.

Other software available for proteomic and interactome analysis can also be used for CP data processing. For instance, free tools such as Perseus (Tyanova et al., 2016b), PrInCE (Stacey et al., 2017) and EPIC (Hu et al., 2019) may provide suitable options for visualization, protein quantification, statistical analysis, prediction of PPIs from co-fractionation data, obtention of supporting info from public repositories and/or cross-omics comparisons.

### 2.5 Storage, Sharing and Examination of Complexome Profiling Data

**FIGURE 2:**
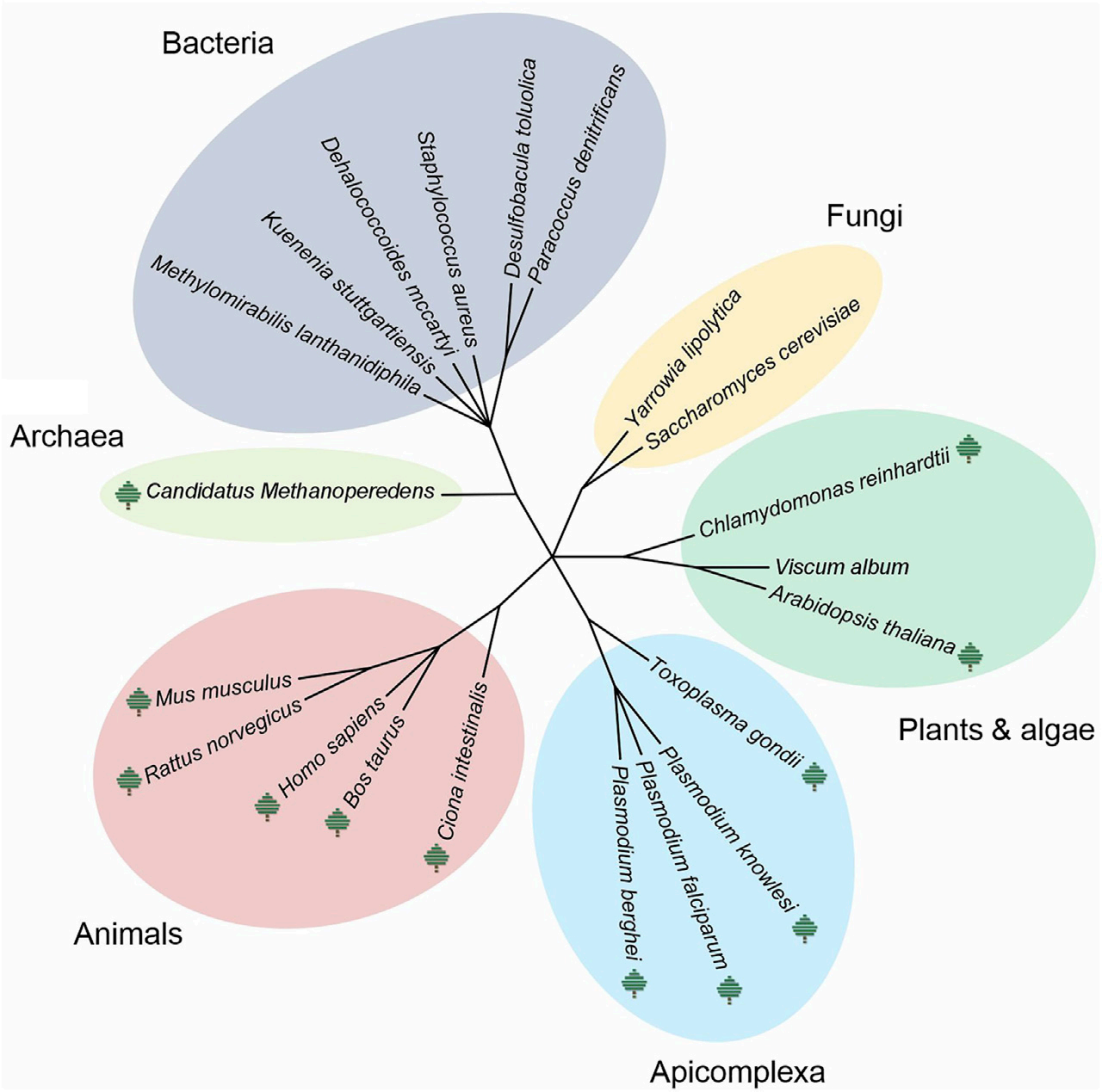
Different species used in complexome profiling studies. Phylogenetic tree including all the species reported in complexome profiling (CP) studies until November 2021. As it can be seen, CP has been used to study multiple species covering the three different life domains. Where a tree-like icon is shown next to the species name, CP data are currently available on CEDAR (https://www3.cmbi.umcn.nl/cedar/browse/). The template of the tree was generated online using Interactive Tree of Life (iTOL) v6.3.3 (https://itol.embl.de/) and further edited manually.

Although CP datasets contain a long list of identified proteins, the majority of studies have focused only on a small subset of the identified protein complexes. Naturally, the amount of information contained in those datasets could be useful not only to unveil uncharacterized PPIs, but also to validate previously reported protein complexes. All these under-explored results thus represent a valuable source of information, which could be reused and further mined by other researchers. To achieve this purpose, the ComplexomE profiling DAta Resource (CEDAR) has recently been established as the first website for depositing and sharing CP data (van Strien et al., 2021). A standardized format containing the minimum information required for a CP experiment (MIACE) ensures compatibility and correct retrieval of data. CEDAR is able to link access to raw MS data and other files deposited parallelly in the known repository PRIDE (ProteomeXchange consortium) (Perez-Riverol et al., 2019). Additionally, CEDAR has a profile viewer tool that allows users to examine CP data directly on the website. Since its release in June 2020, 24 complete CP experiments encompassing a total of 146 samples have been submitted to CEDAR (web V1.1, accessed October 6, 2021). CP data from 11 species covering different life domains are already accessible (Figure 2).

**FIGURE 3:**
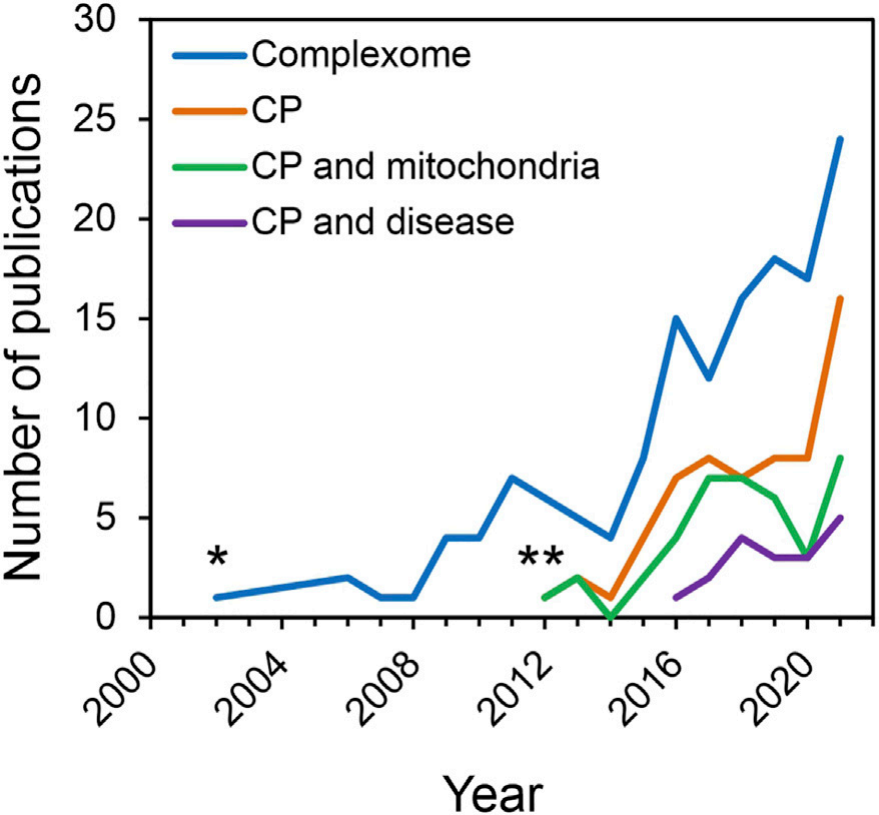
Reported studies in the literature including the complexome profiling approach. Since the introduction of the term “complexome” back in 2002 (*), ∼145 publications referring to this term have been reported in PubMed (NCBI) (blue line). Formal introduction of complexome profiling (CP) in 2012 (**). In less than 10 years, ∼62 studies have applied CP (orange line), where ∼40 and ∼18 publications are related to mitochondrial research (green line) or to protein complexes involved in disease (purple line), respectively. The positive tendency of multiple groups around the world implementing CP to study protein-protein interactions in both fundamental- and disease-related studies as a standard method is incontrovertible.

It should be mentioned that the data available through CEDAR are just a fraction of the CP studies reported in the literature. At present, ∼145 publications mentioning the term “complexome” (or complexomic/complexomics) have been found in PubMed (NCBI) records; ∼62 publications correspond to CP-related studies, reviews, methods and software tools (Figure 3). In ∼40 and ∼18 publications, CP has been used for mitochondrial research or to study protein complexes involved in disease, respectively (Figure 3). As the number of CP publications continues accelerating, it is desirable that future studies using this approach would also share resultant CP datasets publicly through CEDAR to simplify their reuse by the scientific community and keep expanding its valuable offer.

## 3 Complexome Profiling as a Tool to Investigate Mitochondrial Protein Complexes

Mitochondria are traditionally known as the eukaryotes’ “powerhouses” since they contain all the enzymes required to generate ATP via the oxidative phosphorylation (OXPHOS) pathway. This process is catalyzed canonically by four respiratory chain complexes (I, II, III and IV) and a F_1_F_O_-ATP synthase (complex V). Complexes I, III and IV couple the energy released during electron transfer to oxygen to the generation of an electrochemical gradient of protons across the inner mitochondrial membrane (IMM). The proton gradient not only drives the synthesis of ATP, but also other processes such as metabolite transport, protein import, redox and Ca^2+^ homeostasis, fusion/fission, signaling and cell death. These organelles do also contain their own genome, also known as mitochondrial DNA (mtDNA), which encodes for a few but essential subunits of OXPHOS complexes. The roles of mitochondria are thus not limited to their energy duties. These organelles are multi-functional “hubs” critical to almost every cell process.

Despite of the great progress in molecular characterization and structure elucidation of numerous mitochondrial protein complexes, mostly OXPHOS- and mitoribosome-related proteins, a large number of mitochondrial PPIs remain elusive. In the last years, however, CP has proven valuable for identifying novel protein interactors, validating previous results and shedding light on intricate assembly pathways. In this section, we summarize these findings and describe how CP has boosted the analysis of protein complexes in the mitochondrial research field. Expectedly, most of these findings are again related to components or mediators of the assembly of OXPHOS complexes, energy metabolism-linked proteins and mitoribosomes.

The first groups that established CP have also been interested in the many features of OXPHOS and in particular of complex I (CI). CI is the largest redox enzyme of the mitochondrial respiratory chain constituted by ∼45 subunits depending on the species. CI generates proton-motive force driven by the transfer of electrons from NADH to ubiquinone (Hirst, 2013). Although the redox features and composition of mitochondrial CI in several species were already known by the start of the 2010s, major queries on this enzyme had yet unresolved: its entire 3D structure, its energy-conversion mechanism and how it assembles. To help tackle the last one, the formal introduction of CP by Heide and co-workers was useful to identify TMEM126B interacting with the known CI assembly factors NDUFAF1, ECSIT and ACAD9 (Heide et al., 2012). *TMEM126B* knockdown in 143B osteosarcoma cells led to ∼95% specific decrease in CI-containing supercomplexes, i.e., supramolecular associations of complexes I, III and IV. These results thus proposed TMEM126B as a CI assembly factor. In an earlier report using HEK293 cells, Wessels and co-workers identified with a similar approach two other assembly factors of CI: C6ORF66 and C3ORF60 (Wessels et al., 2009), currently known as NDUFAF4 and NDUFAF3, respectively. In this study, mitochondrial proteins were separated by BN-PAGE followed by LC-MS/MS identification, but comparison of migration patterns was limited to the use of PCP.

A few years later, a dynamic CP strategy was successfully implemented to describe the step-by-step integration of the subunits, assembly factors and the different assembly intermediates of human CI (Guerrero-Castillo et al., 2017a). The authors used CP data from time-based mitochondrial translation recovery to describe a number of assembly intermediates of CI accumulating at various timepoints after removal of chloramphenicol, a reversible inhibitor of mitoribosomes (Ugalde et al., 2004). These data corroborated the long time proposed modular assembly pathway of CI, which basically involves the coordinated formation and pre-assembly of its functional modules, N, Q, P_P_ and P_D_, before forming the entire enzyme. This study also provided insight into the specific involvement of earlier reported assembly factors and novel interactors. Complex IV (CIV) assembly-related chaperone COA1 and TMEM186 were clearly found associated with membrane arm intermediates that also interact with TMEM126B, NDUFAF1 and ECSIT. At present, all these proteins have been recognized as true components of the so-called mitochondrial CI assembly (MCIA) complex (Formosa et al., 2020). ATP5SL clustered with FOXRED1, another CI assembly factor. However, ATP5SL disruption did not lead to CI deficiency (Andrews et al., 2013) and its involvement in the assembly pathway is thereby unclear. Complex V assembly-involved protein TMEM70 was also identified in assembly intermediates of CI. To further explore the putative role of TMEM70 on CI assembly, Sánchez-Caballero and co-workers performed a study including proximity-dependent biotin identification (BioID), co-evolution analyses and CP (Sanchez-Caballero et al., 2020). In this study, TMEM70 was found in close contact with subunits of complexes I and V, as well as the small mitoribosome subunit. The absence of TMEM70 resulted in slight depletion of fully assembled CI and accumulation of assembly intermediates of the distal part of the membrane arm of CI, whereas mature complex V (CV) content was ∼70% lower than in the control cell line. These authors showed TMEM70 as a non-essential assembly factor of complexes I and V, and proposed a possible role in tethering mitoribosomes to the IMM during translation.

Apart from studies in human cells, CP has also been used to study the role of several accessory subunits of CI in different models. For example, Kmita and co-workers analyzed the assembly of CI in a deletion strain of the yeast *Yarrowia lipolytica* lacking the Zn^2+^-containing subunit NUMM/NDUFS6 (Kmita et al., 2015). Absence of this subunit did not prevent formation of the entire CI, since a lower enzyme content and higher fraction of peripheral arm subcomplexes were observed. Unexpectedly, the almost fully assembled CI contained the assembly factor N7BML/NDUFAF2 bound instead of accessory subunit N7BM/NDUFAF12. Moreover, Angerer et al. implemented CP in a study on the mitochondrial acyl carrier proteins (ACPM) 1 and 2, which are accessory subunits of CI and contain LYR motifs (Angerer et al., 2014). Although ACPM1/2 are predominantly associated to CI, ACPM1 was also found as a free protein in the matrix and forming a complex with LYRM4(ISD11)/NFS1 involved in iron-sulfur cluster biosynthesis. Furthermore, two different groups in parallel unveiled the existence of two isoforms of the peripheral arm subunit NDUFV3 of CI in mammals (Bridges et al., 2017; Guerrero-Castillo et al., 2017b). An extra exon present in gene *NDUFV3* can be alternative spliced, hence generating short and long isoforms of ∼10 and ∼50 kDa, respectively. CP helped find a different expression of these isoforms in different tissues of bovine, mouse and rat as well as in cultured human cells. The canonical short isoform was predominantly identified in heart and skeletal muscle, whereas the large isoform was the foremost isoform in liver, brain and lung tissues. Both NDUFV3 isoforms can also be expressed at the same time and correctly assembled onto CI in some tissues; yet, one isoform predominated in each case.

Other OXPHOS complexes and their chaperones have also been explored by CP. Singhal et al. identified the product of ORF *YDR381C-A* as an assembly factor of yeast complexes III and IV (Singhal et al., 2017). This protein was renamed as cytochrome *c* oxidase interacting protein 1 (Coi1), which occurs only in fungi. Deletion of Coi1 resulted in severe alteration of mitochondrial function, diminished amounts of CIV-associated heme, defective assembly of complexes III, IV and their supercomplexes, as well as accumulation of assembly intermediates. Vidoni and co-workers reported that the short isoform of myofibrillogenesis regulator 1 (MR-1S) associates with chaperones PET100 and PET117 to mediate human CIV assembly (Vidoni et al., 2017). Authors implemented not only CP to study assembly intermediates accumulated in control and *MT-CO3* mutant cybrids, but also SILAC and quantitative MS.

CP has been helpful to better understand the formation of supercomplexes or respirasomes. The specific factors for mediating this process remain unclear. COX7A2L, also known as SCAFI, has been shown critical for the association of complexes III and IV (Perez-Perez et al., 2016). Its putative involvement in larger respirasome formation has also been reported (Lapuente-Brun et al., 2013). To better understand the role of SCAFI, Férnandez-Vizarra et al. explored the mitochondrial complexome of a SCAFI knockout human cell line by SILAC-based CP (Fernández-Vizarra et al., 2021). Absence of SCAFI resulted in marked loss of supercomplex III_2_-IV, whereas CI-containing respirasomes were not affected. In contrast, authors showed that ∼70% of respirasomes contained COX7A2 in either human cell line analyzed. This discrepancy has apparently been related to a tissue-specific expression of the two isoforms (Lapuente-Brun et al., 2013). On the other hand, Protasoni and co-workers demonstrated that absence of a fully assembled complex III (CIII) in a MT-CYB-deficient human cell line stalled CI biogenesis by preventing the integration of its N-module (Protasoni et al., 2020). Although substantial accumulation of partially assembled Q/P intermediate was observed by SILAC-based CP, a slight fraction of CI could still be detected. Reasonably, respirasome assembly was totally lost in the mutant. Besides, assembly of CIV was affected since several COX subunits were found associated with CIII sub-assemblies; hence interfering their correct assembly. It has thus been proposed that human complexes I, III and IV assemble in a cooperative fashion, where CIII seems to be central.

Mitochondria are characterized by a highly folded IMM. These folds, called cristae, have emerged as dynamic compartments whose shape and dimensions influence structure and functioning of the OXPHOS system (Cogliati et al., 2016b). A key player in shaping cristae appears to be the MICOS complex, which also interacts with the sorting and assembly machinery (SAM) complex from the outer mitochondrial membrane (OMM) (Huynen et al., 2016). Interaction of these two complexes constitutes the mitochondrial intermembrane space bridging (MIB) complex (Ott et al., 2015). Since its relatively recent discovery (Harner et al., 2011), much of what we know about the MICOS complex has been derived from CP-based studies.

For instance, Weber and co-workers identified apolipoprotein O (APOO/MIC26) and apolipoprotein O-like protein (APOOL/MIC27) as potential components of the human MIB complex (Weber et al., 2013). Huynen and co-workers described the composition and apparent masses of the fully assembled MIB complex (2.2–2.8 MDa) and other putative assembly intermediates, e.g., the free MICOS complex (∼700 kDa) (Huynen et al., 2016). Anand et al. identified MIC13 (QIL1) as a novel MICOS complex subunit (Anand et al., 2016). Deletion of MIC13 resulted in a smaller albeit still assembled MICOS complex while having no effect on integrity of OXPHOS complexes. They also demonstrated a disruptive effect of MIC13 deletion on cristae morphology accompanied by reduced respiratory capacity. The same group used a similar approach to characterize MIC26 and MIC27 (Anand et al., 2020). They showed negative effects on CV stability as well as cristae morphology defects both of which were much more pronounced in the double knockout than either single knockout, suggesting overlapping roles. Assembly of other MICOS components was however unimpeded by MIC26/27 knockouts, proposing these proteins are not essential for its assembly or stability. The link of MICOS components to CV was further explored by Eydt et al. (2017), showing that MIC10 comigrates with CV dimers. Additionally, they presented evidence that MIC10 acts antagonistically to MIC27 in negatively controlling CV oligomerization, while MIC27 appears to be a positive regulator. A recent paper by Bock and co-workers reported the interaction of PGC-1- and ERR-induced regulator in muscle 1 (PERM1) with multiple components of the MICOS/MIB complex as well as vimentin and ankyrin B in skeletal muscle from mouse (Bock et al., 2021). These findings suggested a novel mechanism to help understand not only the interconnection of mitochondria and sarcolemma but also mitochondria-cytoskeleton associations as well as the organization of a functional mitochondrial network in this tissue. Taken together CP has greatly aided our understanding of MICOS/MIB complex composition, the role of individual subunits and its interaction with OXPHOS complexes.

The IMM contains other key protein complexes involved in proteolytic events, fusion/fission of mitochondrial membranes, regulation of cristae morphology and cell signaling. Some of these proteins belong to the SPFH (stomatin, prohibitin, flotillin and HflC/K) family and usually arrange as large scaffolds. To have a clearer picture, Wai and co-workers implemented CP to unveil the interaction partners of stomatin-like protein 2 (SLP2) and at least two proteases, PARL and YME1L, in immortalized embryonic fibroblasts mitochondria (Wai et al., 2016). SLP2 works as a regulator of PARL, which also modulates other proteins such as PGAM5, PINK1 and OMA1. These interactions were suggested to occur in defined sites of the IMM and related to mitochondrial proteostasis, dynamics and cell survival. Similarly, Konig and co-workers identified MAIP1 (C2Orf47) as a novel *m*-AAA protease-binding protein in complexome profiles (Konig et al., 2016). MAIP1 was shown essential for regulating the assembly of subunit EMRE into mitochondrial Ca^2+^ uniporter (MCU) complexes.

CP has also played an important role in the field of plant and parasite mitochondrial biology. Using CP, Senkler and co-workers performed a systematic characterization of mitochondrial complexes in the plant *A. thaliana* (Senkler et al., 2017). CP data were also useful to update the subunit composition of OXPHOS complexes and identifying respective assembly intermediates. This group then used the same approach on mitochondria from the mistletoe *V. album*, revealing a highly unusual OXPHOS system (Senkler et al., 2018). This species completely lacks CI, the only multicellular eukaryote to date with this distinction; instead, *V. album* contains alternative NAD(P)H oxidoreductases. This species also expresses an alternative oxidase and its complexes III and IV are firmly associated as supercomplexes. In comparison to *A. thaliana*, the abundance of complexes II and V was particularly low, suggesting a shift in stoichiometry of the OXPHOS complexes in the IMM of this plant. Rugen and co-workers took a more focused CP strategy to investigate the composition of the mitoribosome in *A. thaliana* (Rugen et al., 2019). Utilizing LP-BN-PAGE- and DGU-based CP setups, several non-conventional proteins were found attached to the mitoribosome, mostly from the class of pentatricopeptide repeat proteins that seem to be involved in RNA processing and protein maturation. Presence of these additional interactors results in a larger mitoribosome with unusual large and small subunits of ∼3 and ∼5.7 MDa, respectively. Additional functions are thus likely incorporated into the plant mitoribosome.

Due to their extreme divergence, apicomplexan parasites of the genera *Plasmodium* and *Toxoplasma* that cause the infectious diseases malaria and toxoplasmosis, respectively, remain poorly understood. In fact, more than one-third of their genes still lack any functional annotation (Aurrecoechea et al., 2009; Harb et al., 2020). To help narrow this gap, Evers et al. and Maclean et al. obtained evidence for highly divergent composition of the mitochondrial OXPHOS complexes in *Plasmodium falciparum* and *Toxoplasma gondii*, respectively (Evers et al., 2021; Maclean et al., 2021). More than 30 novel subunits across complexes II, III, IV and V with no recognizable orthologs outside of the myzozoan phylum could be identified by CP. These novel subunits did not only replace subunits typically observed in standard models, but also increased the size of *P. falciparum* OXPHOS complexes by around 50, 50, 130 and 70% as compared to complexes II, III, IV and V from mammalian mitochondria, respectively. This was consistent with the observations in *T. gondii*, thus making apicomplexan OXPHOS complexes the largest described to date. Furthermore, abundance of OXPHOS complexes was ∼32 fold increased in the transmissible gametocyte stages compared to the pathogenic asexual stages of *P. falciparum* (Evers et al., 2021). This finding offered protein-level support for the long-standing hypothesis that malaria parasites undergo a metabolic switch towards mitochondrial catabolism to facilitate their transmission back to the insect vector (MacRae et al., 2013). Despite its relatively recent introduction to the field of parasite research, CP has already provided valuable data for mapping a number of previously unknown interactors to protein complexes and biological processes. As these novel additions are unlike what is known from standard models, it opens the door for new discoveries, even in pathways as fundamental as respiration.

## 4 The Mitochondrial Complexome and Human Disease

In the last years, several metabolic diseases have been characterized along with accompanying complexome studies of human subjects carrying mutations in subunits of the OXPHOS complexes, assembly factors or proteins required for efficient mitochondrial protein biosynthesis. Most of these studies involved patients with metabolic disorders that affect mitochondrial processes leading to heterogeneous clinical manifestations ranging from mild to severe phenotypes. These studies analyzed subcellular preparations from primary skin fibroblasts from patients and controls, or from cell lines with genetic mutations/deletions used as disease models. CP analysis revealed either changes in abundance of specific protein complexes, indicating alterations of their relative steady-state levels in comparison to the controls, or molecular mass shifts resulting from the loss of protein components or from destabilization of the complexes. Beyond the described main alteration of the complex directly involved with the respective genetic mutation, an enormous amount of information can be retrieved from the deposited complexome data of these studies that could potentially be re-analyzed and investigated, for instance, in relation to the clinical features of these patients. Such in-depth characterization of the mitochondrial proteome at the level of macromolecular complexes in disease states may conceivably help uncover molecular basis and consequences of metabolic disorders and, importantly, could serve to identify molecular targets for developing novel therapeutic strategies. In this section, we summarize studies of genetic defects causing metabolic diseases in human subjects and mutations in model organisms analyzed to date by CP (Table 1).

**TABLE 1:** Complexome profiling studies in biomedical research.

Gene	Disease/Impairment	OMIM^a^	Clinical features/Phenotype		Pathway/Mechanism	Effect of mutation	Sample type	CEDAR/PRIDE entry^b^	References
Mitochondrial CI deficiency									
*TMEM126B*	CI deficiency	# 618250	Muscle weakness, elevated lactate and alanine		Assembly of CI, peripheral arm, P-proximal ND2-module	Accumulation of assembly intermediates of CI	Patient fibroblasts	n.d.	Alston et al. (2016), Sánchez-Caballero et al. (2016b), Fuhrmann et al. (2018)
*NDUFAF8*	Leigh Syndrome, CI deficiency	# 618776	Encephalopathy, seizures, neuroradiologic features		Assembly of CI, Q-module (?)	Decreased CI activity and abundance, impaired assembly of the Q-module	Patient fibroblasts	CRX15	Alston et al. (2020)
*NDUFC2*	Leigh Syndrome, CI deficiency	# 619170	Developmental regression, lactic acidosis, neurologic deterioration		CI subunit, P-proximal ND2-module	Accumulation of assembly intermediates of CI	Patient fibroblasts	PXD014936	Alahmad et al. (2020)
*NDUFA6*	CI deficiency	# 618253	Delayed development, neurologic deterioration, optic atrophy		CI subunit, Q-module	Destabilization of the Q-module of CI	Patient fibroblasts	n.d.	Alston et al. (2018)
*TMEM70*	CV deficiency	# 614052	Mitochondrial encephalocardio-myopathy, 3-methylglutaconic aciduria, CV deficiency		Assembly of CI and CV	Decreased CI and CV activities, accumulation of subassemblies of CI and CV	HAP1, TMEM70-KO	CRX10	Sanchez-Caballero et al. (2020)
*DNAJC30*	LHON	# 619382	Bilateral vision loss		CI maintenance	Slower turnover of the N-module subunits	Patient fibroblasts, HEK293, DNAJC30-KO	PXD021385	Stenton et al. (2021)
								PXD021386	
								PXD021500	
								PXD022340	
								PXD022339	
								PXD021548	
								PXD021499	
*CLPP*	Perrault syndrome	# 614129	Sensorineural hearing loss, premature ovarian failure		CI turnover	—	HEK293-KO, MEF-KO, Mouse heart-KO	CRX12	Szczepanowska et al. (2020)
Mitochondrial CIII deficiency									
*MT-CYB*	LHON	* 516020	Weakness, ataxia, neurologic involvement; combined respiratory chain deficiency		CIII subunit	Decreased enzyme activities of CI, CIII and CIV	143B-derived cybrid cells	CRX19, CRX26	Protasoni et al. (2020), Palenikova et al. (2021b)
*TTC19*	Mitochondrial complex III deficiency nuclear type 2 (MC3DN2)	# 615157	Motor disability with ataxia, apraxia, dystonia and dysarthria. Cognitive impairment and axonal neuropathy		CIII maturation	Decreased CIII activity due to failure to remove inhibitory N-terminal fragments of UQCRFS1	Mouse brain mitochondria	n.d.	Bottani et al. (2017)
Mitochondrial CIV deficiency									
*COX4I1*	CIV deficiency	# 619060	Failure to thrive, neurologic involvement, hypotonia, seizures, cerebellar atrophy		CIV subunit	Impaired assembly of CIV and CI	HEK293-KO	CRX25	Cunatova et al. (2021)
*COX4I2*	Exocrine pancreatic insufficiency, dyserythropoietic anemia, and calvarial hyperostosis	# 612714	Steatorrhea, anemia, failure to thrive		CIV subunit	Impaired assembly of CIV and CI	HEK293-KO	CRX25	Cunatova et al. (2021)
*MT-CO1*	LHON, Mitochondrially inherited non-syndromic sensorineural deafness	# 535000, # 500008, * 516030	Heterogeneous variety of neuromuscular disorders		CIV subunit	Loss of CIV holoenzyme; destabilization of CI-CIII_2_ supercomplex (S_0_)	143B-derived cybrid cells	CRX17	Lobo-Jarne et al. (2020)
*MT-CO2*	CIV deficiency	* 516040	Progressive gait ataxia, cognitive impairment, bilateral optic atrophy, pigmentary retinopathy		CIV subunit	Loss of CIV holoenzyme	143B-derived cybrid cells	CRX17	Lobo-Jarne et al. (2020)
*COX7A2L*	—	* 605771	—		Stabilization of SCs containing CIII_2_ and CIV	Aberrant supercomplexes formation (?)	HEK293, SCAFI-KO	CRX28	Fernández-Vizarra et al. (2021)
Combined OXPHOS deficiency									
*MRPS2*	Combined OXPHOS complexes deficiency	# 617950		Mild multisystem disease; Sensorineural hearing loss, hypoglycemia; Mitochondrial dysfunction	Component of the 28S mitochondrial ribosomal subunit	Decreased abundance of OXPHOS complexes, and destabilization of the 28S mitochondrial ribosomal subunit	Patient fibroblasts	CRX22	Gardeitchik et al. (2018)
*MRPS22*	Combined OXPHOS complexes deficiency; ovarian dysgenesis	# 611719, # 618117		Cardiomyopathy, metabolic acidosis, reduced mitochondrial OXPHOS complexes activities	Component of the 28S mitochondrial ribosomal subunit	Decreased abundance of OXPHOS complexes, and destabilization of the 28S mitochondrial ribosomal subunit	Patient fibroblasts	CRX22	Gardeitchik et al. (2018)
*TAZ*	Barth syndrome	# 302060		3-methylglutaconic aciduria, neutropenia, dilated cardiomyopathy, skeletal myopathy	Cardiolipin remodeling/Membrane curvature	Widespread reorganization of the mitochondrial complexome	Patient fibroblasts	CRX8	Chatzispyrou et al. (2018)
*METTL15*	—	* 618711		—	N4-methylcytidine methyltransferase	Decreased mitochondrial protein biosynthesis	HAP1-KO HeLa	CRX13	Van Haute et al. (2019)
*ERAL1*	Perrault syndrome	# 617565		sensorineural deafness, ovarian dysgenesis	28S mitochondrial ribosomal subunit assembly	Decreased 28S mitochondrial ribosomal subunit abundance	Patient fibroblasts	n.d.	Chatzispyrou et al. (2017)
mtDNA loss	—	—		—	OXPHOS complexes I, III, IV, V, and t- and rRNAs	Dysfunctional mitochondrial energy metabolism	Rho0 cells	CRX32	Guerrero-Castillo et al. (2021b)
Other									
*CLPB*	MEGCANN	# 616271		3-methylglutaconic aciduria, neutropenia, neurologic symptoms	Intermembrane space ATP-dependent dissagregase	Aberrant interaction between CLPB and HAX1	Patient fibroblasts	n.d.	Wortmann et al. (2021)
*ATP6V1A*	Cutis laxa, developmental and epileptic encephalopathy	# 617403, # 618012		Wrinkled skin, hypotonia, dysmorphic facial features, cardiovascular and neurologic involvement	V-type ATPase subunit, V_1_ segment	Destabilization of V-type. ATPase holocomplex	Patient fibroblasts	CRX11	Van Damme et al. (2017)
*ATP6V1E*	Cutis laxa	# 617402		Wrinkled skin, hypotonia, cardiopulmonary involvement	V-type ATPase subunit, V_1_ segment	Destabilization of V-type, ATPase holocomplex	Patient fibroblasts	CRX11	Van Damme et al. (2017)
*ATP6V0A2*	Cutis laxa	# 219200		Wrinkly skin syndrome; varying degrees of growth and developmental delay and neurologic abnormalities	V-type ATPase subunit, V_O_ segment	Decreased abundance of V-type ATPase holocomplex	Patient fibroblasts	CRX11	Van Damme et al. (2017)
*PI4K2A*	Cutis laxa	* 609763		Wrinkled skin, choreoathetoid movement disorder, dysmorphic features and intellectual disability	Phosphoinositide lipids regulation	Impaired intracellular signaling and trafficking	Patient fibroblasts	n.d.	Mohamed et al. (2020)
*PEX19*	Zellweger syndrome	# 614886		Hypotonia, seizures, craniofacial anomalies, neuronal migration defects, hepatomegaly, chondrodysplasia punctate	Peroxisome biogenesis	Impaired import of peroxisomal membrane proteins	Wild-type yeast and single and double deletion strains for PEX19 and ATAD1 yeast orthologs, *pex19*Δ and *msp1*Δ, respectively	PXD024625	Nuebel et al. (2021)

a Online Mendelian Inheritance in Man® (OMIM); #: Phenotype descriptive entries, *: Gene entries.

b CEDAR entries correspond to CRX codes, whereas PRIDE (ProteomeXchange) entries correspond to PXD codes.

Mitochondrial OXPHOS complexes are shown as: CI, complex I; CIII or CIII_2_, complex III; CIV, complex IV and CV, complex V. Abbreviations; KO, knockout; MEGCANN, 3-methylglutaconic aciduria, type VII, with cataracts, neurologic involvement and neutropenia; LHON, Leber hereditary optic neuropathy; n.d., not deposited.

### 4.1 Complex I Deficiency

CI deficiencies may potentially originate from genetic defects in either the seven genes encoded in the mtDNA or in the 37 subunits and the increasing number of assembly factors encoded in the nuclear DNA (Fassone and Rahman, 2012; Sánchez-Caballero et al., 2016a). Pathogenic mutations in patients have been found in the majority of CI subunits and in many assembly factors (Rodenburg, 2016), and the number of mutations identified continuously grows. Since TMEM126B was the first assembly factor of CI identified by CP, it was not unexpected that the first complexomes from patient fibroblasts with genetic alterations described in the literature belonged to subjects harboring mutations in this gene. Independently, two research groups described six (Alston et al., 2016) and three (Sánchez-Caballero et al., 2016b) subjects, respectively, presenting isolated CI deficiency with muscle weakness in most cases and with recessive mutations, evidenced by whole exome sequencing, in the gene encoding TMEM126B. Complexome profiles of three subjects revealed aberrant migration patterns of CI subunits with decreased abundance of mature CI-containing supercomplexes at ∼1.7 MDa and accumulation of assembly intermediates. In both studies, increased steady-state levels of subunits belonging to the Q-module plus assembly factors NDUFAF3 and NDUFAF4, and to the P-distal ND4 module indicated stalled assembly intermediates of these unaffected modules, suggesting that TMEM126B takes part, together with the other MCIA complex components, ACAD9, NDUFAF1 and ECSIT, in the assembly of the P-proximal ND2 module. Complementation of patient fibroblasts using lentiviral expression of wild-type TMEM126B partially restored the abundance of CI-containing supercomplexes and decreased the abundance of Q- and P-module assembly intermediates, validating its role as assembly factor. Notably, specific ubiquitination and further degradation of this assembly factor has been implicated in a regulatory mechanism to decrease CI content and consequently oxygen consumption under hypoxic conditions (Fuhrmann et al., 2018).

Complexome profiles of patient fibroblasts with mutations either in a subunit or in an assembly factor of the Q-module, NDUFA6 and NDUFAF8, respectively, proved useful to corroborate the pathogenicity of the respective mutation and for pointing out the stage in which CI assembly was stalled. Bi-allelic mutations in NDUFA6, a LYR-motif subunit important for proper functioning of ubiquinone redox chemistry (Galemou Yoga et al., 2020), were found responsible for defects in CI assembly (Alston et al., 2018). In one of the three subjects studied, it has been found a decrease not only in the subunits of the Q-module, NDUFA6, NDUFA7 and NDUFA12, but also in subunits belonging to the N-module, correlating with the more severe phenotype observed in this subject. Of note, in these three patients, assembly factor NDUFAF2, which is known to interchange for subunit NDUFA12 in a late assembly step (Pereira et al., 2013), remained to some extent bound to CI intermediates, indicating that also in the other two subjects N-module incorporation was inefficient. On the other hand, NDUFAF8 has been identified to physically interact with NDUFAF5 (Floyd et al., 2016), an assembly factor of CI required to hydroxylate an arginine residue of the Q-module subunit NDUFS7 (Rhein et al., 2016). Alston and co-workers identified three subjects presenting Leigh syndrome with bi-allelic mutations in NDUFAF8 causing isolated CI deficiency (Alston et al., 2020). Complexome profile of one of the patients revealed decrease abundance of CI-containing supercomplexes. Consistent with the role of this protein in early CI assembly, NDUFAF8-mutated patient fibroblasts showed incomplete formation of the Q-module since neither its signal nor the fraction of this module holding assembly factors NDUFAF3/4 were observed. These fractions are visible even in control cells where CI assembly process occurs normally; in particular the more prominent one including the assembly factors. In contrast, accumulation of the earliest Q-module sub-assembly formed only by subunits NDUFA5, NDUFS2 and NDUFS3 has been described. In addition, accumulation of the P-distal ND4 module has also been reported.

Extending the genotype of CI deficiency, pathogenic mutations in subunit NDUFC2 have been identified in two unrelated children presenting Leigh syndrome (Alahmad et al., 2020). CP analysis from both subjects indicated severe impairment of CI assembly since abundance of CI-containing supercomplexes decreased prominently. In addition, intermediates composed by subunits of the Q-module, the Q/P-proximal module and the P-distal ND4 module accumulated, suggesting that stalling of CI assembly in these patients occurred at the intermediate stage (Guerrero-Castillo et al., 2017a). Comparably, mutations in the gene encoding TMEM70 have been identified in patients with deficiencies in complexes I and V (Jonckheere et al., 2011). The importance of TMEM70 in CV assembly has become clear from many mutations identified in human subjects (Cízková et al., 2008; Honzík et al., 2010), but recently in-depth analyses of complexome profiles delineated the role of TMEM70 in the formation of the F_1_-c intermediate (Sanchez-Caballero et al., 2020). In regard to CI however, accumulations of assembly intermediates corresponding to the Q/P-proximal module as well as to the P-distal ND5 module, evidenced by complexome profiling of TMEM70 knockout HAP1 cells, indicated stalling of CI assembly at the binding of P-distal ND4 module to these intermediates (Sanchez-Caballero et al., 2020).

CI deficiency may not exclusively originate from pathogenic mutations in structural components and assembly factors, but also from mutations in genes involved in the maintenance and repair of damaged subunits. In an exhaustive study including 29 families (Stenton et al., 2021), Stenton and co-workers identified mutations in DNAJC30 as a cause of LHON syndrome. Co-migration of DNAJC30 with OXPHOS supercomplexes was identified in the control but not in the patient complexome profile. The abundance of CI assembled into supercomplexes, slightly higher in the patient samples, excluded a role of DNAJC30 in the assembly of CI. Instead, turnover of subunits of the N-module of CI that are more frequently interchanged during optimal maintenance was slowed down, implicating DNAJC30 in a repair mechanism of CI still associated into the supercomplex. Interestingly, the ATP-dependent serine protease CLPP, whose mutations in humans are known to cause Perrault syndrome (Jenkinson et al., 2013), has also been implicated in CI repair by selectively replacing subunits of the N-module that are exposed to oxidative damage (Szczepanowska et al., 2020).

### 4.2 Complex III Deficiency

Proper assembly of CIII is not only important for generation of proton-motive force driven by the enzymatic oxidation of ubiquinol molecules produced by multiple dehydrogenases localized in the IMM, but also, at the core of respiratory supercomplexes, for structural stabilization of complexes I and IV. This phenomenon has been observed in several CIII-deficient patients where the activity and content of CI and CIV were concomitantly decreased (recently reviewed by Vercellino and Sazanov, 2021). In addition, a SILAC-duplexed CP study of cells lacking subunit cytochrome *b* due to a 4-bp deletion in *MT-CYB* and therefore unable to form holo-complex III, was consistent with a role of CIII-containing supercomplexes in CI maturation, since a late assembly intermediate containing assembly factor NDUFAF2 accumulated in cells with mutated cytochrome *b* (Protasoni et al., 2020). In contrast, mutations that partially affect the enzymatic activity of CIII, but not its assembly, do not compromise the stability and activity of CI and CIV. For instance, using a mouse model of CIII deficiency by deletion of TTC19, a protein involved in the removal of inhibitory proteolytic fragments of the Rieske iron-sulfur protein from the holo-complex, Bottani and coworkers showed that while the activity of CIII decreased in the tissues tested, the content and activity of CI and CIV were hardly altered (Bottani et al., 2017).

### 4.3 Complex IV Deficiency

Respiratory complex IV is a terminal oxidase that transfers electrons from soluble cytochrome *c* to oxygen, which also generates proton-motive force. Although no complexomes from patients with mutations in subunits or assembly factors of CIV have been described so far, multiple cell lines representing disease models of its deficiency have lately been characterized by CP. A complexome study of COX4I1/2 double knockout using HEK293 cells illustrated the interdependency of OXPHOS complexes in terms of their assembly and stability (Cunatova et al., 2021). The lack of subunits COX4I1/2 completely abolished formation of CIV. This is consistent with the role of this subunit forming an early assembly intermediate with COX5. In the absence of CIV, a pronounced decrease in the abundance of the CIV-containing supercomplexes have certainly been evidenced, whereas supercomplex I-III_2_ (S_0_) and complexes II, III and V remained unaffected. Notably, even if complex V forms large macromolecular assemblies that are independent of respirasomes, its interconnection to other OXPHOS complexes became apparent by the accumulation in COX4I1/2 double knockout cells not only of the F_1_ segment, but also of the early F_O_ intermediate containing subunits ATP5MF, ATP5PB, ATP5ME and ATP5MG.

Similarly, Lobo-Jarne and co-workers analyzed the effects of the lack of CIV holoenzyme on the stability of respiratory complexes and respirasomes using trans-mitochondrial cybrids of human osteosarcoma 143B cells harboring nonsense mutations in mitochondrial genes *MT-CO1* and *MT-CO2* that resulted in truncated versions of subunits COX1 or COX2, respectively (Lobo-Jarne et al., 2020). In contrast to control 143B cells that, besides monomeric CIV, contained larger assemblies of this complex corresponding to IV_2_, III_2_-IV and respirasome I-III_2_-IV (S_1_), in cybrids with truncated COX1 and COX2 no CIV was formed but S_0_ remained stable. In the absence of COX1, the only detected assembly intermediate contained subunits COX4 and COX5, whereas the lack of signals of all other unassembled CIV subunits indicated their degradation. Conversely, cells lacking COX2 displayed, in addition to the heterodimer intermediate formed by COX4 and COX5, a larger intermediate containing also subunit COX1. Importantly, these authors described the formation of a heavier S_0_ containing several subunits and assembly factors of CIV, which has also been corroborated by bidirectional co-IP assays. The association of this partially assembled unconventional intermediate of CIV bound to S_0_ exemplified how alternative biogenesis of respirasomes could occur in disease-related conditions.

### 4.4 Combined Oxidative Phosphorylation Complexes Deficiency

The intricate assembly process of OXPHOS complexes requires not only expression and import of functional subunits as well as ancillary proteins produced by cytosolic ribosomes, but also proper expression and insertion of the mtDNA-encoded components. Therefore, diseases featured by energy metabolism alterations may also result from mitochondrial translation deficits. In mammals, the 13 mtDNA-encoded subunits of OXPHOS complexes are synthesized by the mitochondrial translation machinery (Hällberg and Larsson, 2014). This includes the small and large subunits of the mitoribosome [mt-SSU (28S) and mt-LSU (39S), respectively], a set of tRNAs and many regulatory proteins required for the proper functioning and fine tuning of this process (Kummer and Ban, 2021). Taking into account all the factors involved in mitochondrial translation, it is thus not surprising that an increasing number of genetic mutations causing multiple OXPHOS complex deficiencies due to impaired mitochondrial protein biosynthesis have been identified (Pearce et al., 2013).

Perrault syndrome is a rare genetic mitochondrial disorder with a relatively mild phenotype characterized by sensorineural deafness and ovarian dysgenesis. Genetic mutations in several genes encoding mitochondrial and peroxisomal proteins have been identified as direct causes for this disease, including CLPP, HSD17B4, LARS2, HARS2 and TWNK. Chatzispyrou et al. identified two unrelated female patients presenting Perrault syndrome symptoms where whole exome sequencing revealed no mutations in any of the enlisted genes causing this syndrome (Chatzispyrou et al., 2017). Instead, a predicted pathogenic mutation in ERAL1, a chaperone of the 12S mitochondrial rRNA was identified. Complexome profiles of the two patients evidenced a pronounced decrease in the abundance of the mt-SSU, while the mt-LSU remained unaffected; however, very slight changes in OXPHOS complexes intensities have been detected. This correlated with the milder phenotypes of Perrault syndrome patients, contrary to the clinical features observed when the mutations occurred in structural components of the mitoribosome, where multiple OXPHOS complex deficiencies manifested (Gardeitchik et al., 2018).

A study of two subjects with mutations in MRPS2, a structural component of the mt-SSU, has linked these mutations to combined OXPHOS complex defects with sensorineural hearing loss (Gardeitchik et al., 2018). The complexome profile of one of the subjects has been analyzed in parallel with fibroblasts from a previously studied patient with mutations in MRPS22 exhibiting similar symptoms (Smits et al., 2011). Complexomes of both patients revealed a marked decrease in the mt-SSU, while the large subunit remained unaffected. Decrease of mt-SSU abundance was also reflected in the patterns of OXPHOS complexes, which, in both patients, showed largely decreased abundance, particularly for CI and CIV. The abundance of monomeric CV remained unaltered in fibroblasts with mutations in MRPS2 or MRPS22. However, accumulations of assembly intermediates of CV, corresponding to the F_1_ segment and to F_O_ early subassemblies that are not detected in control cell lines also indicated mitochondrial translation impairment in these patients.

A comparable decrease in the abundance of the mt-SSU has been found in a knockout cell line of METTL15, a N4-methylcytidine methyltransferase responsible for modification of the 12S mitochondrial rRNA (Van Haute et al., 2019). The absence of METTL15 impaired the translation of OXPHOS complex subunits encoded in the mtDNA and decreased the steady-state levels subunits of complexes I, III and IV. The DGU-based complexome profiles showed an interaction of METTL15 with mitoribosome components. In contrast to the aforementioned genetic defects where only the mt-SSU was affected, deletion of METTL15 resulted in a parallel decrease of the mt-LSU as well.

Shifting the energy metabolism from oxidative to glycolytic involves remodeling of biochemical pathways and structural rearranges of protein complexes inside and outside mitochondria. In this regard, Rho 0 (ρ^0^) cells, devoid of mtDNA, are incapable to respire due to the lack of essential subunits of OXPHOS complexes thus depend entirely on anaerobic ATP synthesis (King and Attardi, 1989). These cells represent an optimal system to study mtDNA-related defects and OXPHOS deficiencies. Beyond the impaired assembly of OXPHOS complexes I, III, IV and V, which did retain partially assembled intermediates formed by nuclear-encoded subunits, complexomes of ρ^0^ cells displayed unexpected modifications in migration patterns of a wide range of membrane-embedded and water-soluble protein complexes (Guerrero-Castillo et al., 2021b). Changes in expression of, for example, mitochondrial inner membrane carrier proteins, TCA cycle enzymes, mtDNA maintenance proteins and protein translocases localized to both mitochondrial membranes were described. Moreover, the absence of the mtDNA-encoded 12S and 16S rRNAs hindered the formation of mitochondrial ribosomal subunits, from which only smaller parts, composed exclusively of proteins, were preserved. This extensive re-organization of the mitochondrial complexome greatly illustrated the adaptability of mitochondria to self-adjust and compensate the absence of aerobic energy metabolism.

### 4.5 Pathogenic Mutations of Mitochondrial Proteins Affecting Indirectly the Assembly or Stability of Oxidative Phosphorylation Complexes

Reorganization of the mitochondrial energy metabolism can be triggered not only by genetic defects in OXPHOS complexes, assembly factors, mitochondrial translation and/or import machineries, but also by changes in the lipid environment of the inner mitochondrial membrane. This is particularly evident in patients with Barth syndrome, an inherited disorder of cardiolipin remodeling characterized by cardiomyopathy, neutropenia, 3-methylglutaconic aciduria and growth retardation (Neustein et al., 1979; Barth et al., 1983). This syndrome is caused by mutations in *TAZ* (Bione et al., 1996), encoding a mitochondrial acyltransferase called tafazzin, responsible for a CoA-independent exchange of (poly)unsaturated acyl chains from phosphatidyl-choline or phosphatidyl-ethanolamine to cardiolipin (Xu et al., 2006). The polyunsaturated acyl chains shape cardiolipin into a conical form, required for stabilization of membrane curvatures that optimize bioenergetics functions and are especially abundant at the inner mitochondrial membrane of multiple tissues, including heart. Complexome profiles of four Barth syndrome patients with altered saturation patterns of cardiolipin acyl chains revealed marked destabilization of several protein complexes involved in different mitochondrial processes. Significant disruption of supercomplexes containing 2–4 copies of CIV (I-III_2_-IV_2–4_), but not supercomplexes S_0_ and S_1_, has been found as well as partial destabilization of the complexes α-ketoglutarate dehydrogenase and branched-chain α-keto acid dehydrogenase (Chatzispyrou et al., 2018). Importantly, protein complexes involved in maintenance of mitochondrial morphology (e.g., MICOS) and in protein-driven mitochondrial apoptosis were substantially increased in patient fibroblasts. Overall, more than 200 proteins displayed altered migration patterns as a consequence of the membrane curvature destabilization, indicating a widespread reorganization of the mitochondrial complexome. These changes have not been limited to components of the inner mitochondrial membrane; but unexpectedly also spread among complexes located in the other three mitochondrial compartments.

In-depth understanding of remodeling of the mitochondrial complexome might be helpful to explain the links between the biochemical and clinical features observed in patients. For example, to delineate the molecular mechanisms underlying 3-methylglutaconic aciduria or neutropenia. These two clinical signs are also present in subjects with MEGCANN (3-methylglutaconic aciduria with cataracts, neurologic involvement and neutropenia), which is caused by mutations in CLPB, an ATP-dependent refoldase (dissagregase) of the mitochondrial intermembrane space. In a recent study comparing two subjects with bi-allelic mutations in *CLPB* and three subjects with mono-allelic mutations, different patterns of oligomeric CLPB and HAX1, whose genetics variants have been associated to neutropenia, were interpreted as a retention of HAX1 in inefficient CLPB oligomers in the patients (Wortmann et al., 2021). Besides this effect, aberrant migration patterns of multiple mitochondrial proteins were noticed. Comprehensively re-analysing these complexome datasets together with those from Barth syndrome patients and TMEM70 knockout cells, in which accumulation of 3-methylglutaconic acid is commonly observed, could potentially shed light into the causes and consequences of this type of aciduria.

## 5 Complexome Profiling: Perspectives

The implementation of CP analysis to help tackle the many open questions regarding PPIs is not limited to mitochondrial biology. CP is swiftly spreading to other scientific fields and already utilized to investigate protein complexes from other cell compartments and different species. However, there are still key limitations in the current setups, which open the door for further development and expanding its reach. In this final section we discussed not only the most recent advances in CP, but also its application to study protein complexes beyond the mitochondrion.

### 5.1 Improving Separation of Protein Complexes, Mass Spectrometry Quantification and Data Acquisition Time

The biochemical native separation techniques used to date for complexome analysis cover wide molecular mass ranges. Although each of these methods can easily be adapted to enhance the separation resolution of a certain mass window of interest, alternative strategies or modifications to the currently applied methods are envisioned, especially to improve separation resolution of large complexes.

Replacement of detergents by amphiphilic polymers (amphipols) could also help improve the analysis of membrane protein complexes. Amphipol-enclosed transmembrane complexes are more stable during separation than the ones solubilized with detergents (Althoff et al., 2011). Moreover, field flow fractionation (FFF) is a method to separate large (>1 nm) protein complexes. Proteins in solution are passed through a long narrow channel without a stationary phase and separated by their mobility upon a hydraulic field applied perpendicularly (Reschiglian and Moon, 2008), where small particles migrate faster than the bigger ones due to their smaller hydrodynamic sizes and higher diffusion coefficients. This technique has proven effective for analyzing ribosome profiles of the plant *Nicothiana benthamiana* with a similar resolution to the one of density gradients to distinguish ribosomal subunits from monosomes and free proteins (Pitkanen et al., 2014). FFF could thus represent an alternative separation technique in future CP setups.

Resolution of complexome profiles might be significantly improved by increasing the number of fractions in native-PAGE- and SEC-based CP setups. The more fractions the more MS data acquisition time is required however. Hence, a major limitation of CP is LC-MS time; especially if analysis of multiple experimental conditions is intended. Duplexing and triplexing with conventional SILAC/SILAM as well as multiplexing using isobaric labeling compounds are promising strategies for increasing the throughput of CP while reducing MS measuring times (Guerrero-Castillo et al., 2021a; Palenikova et al., 2021b). Incorporation of these strategies in CP studies also offers a higher level of proteomic quantification.

On the one hand, SILAC/SILAM-based quantifications enable more precise comparisons between samples or conditions than label-free quantification methods, and software tools have already been developed to facilitate data analysis (Palenikova et al., 2021a). SILAC is a suitable, affordable and compatible method with practically any cell culture condition. Accuracy and reproducibility are not hampered in this method since there are no precursor interferences as it occurs in isobaric tagging strategies (Chen et al., 2015). Other related variants, such as pulsed (p)SILAC and neutron-encoding (NeuCode) SILAC/SILAM, may also expand the possibilities this strategy can be used for in combination with CP. In pSILAC, the labeled essential aminoacids are added only for a brief period of time during cell growth to monitor differences in newly synthesized proteins and their half-life (Schwanhausser et al., 2009). As aforesaid, pSILAC and CP have proven useful to study the roles of mitochondrial protease ClpXP in turnover of N-module subunits of CI (Szczepanowska et al., 2020) and the chaperone DNAJC30 in the repair mechanism of impaired CI (Stenton et al., 2021). Furthermore, NeuCode SILAC/SILAM allows higher sensitivity and multiplexing with metabolic labeling using heavy stable isotopologues with additional neutrons (Hebert et al., 2013; Merrill et al., 2014; Overmyer et al., 2018). The subtle mass differences caused by neutron-binding energy variations are revealed under ultrahigh-resolution analysis that is only offered by Fourier-transform (FT)-MS or orbitrap analyzer systems (Overmyer et al., 2018). Thereby, implementation of this strategy in CP setups seems plausible.

Multiplexed CP (MCP), on the other hand, employs a set of isobaric labeling compounds, such as tandem mass tags that allows pooling a higher number of samples together after tryptic digestion. With commercially available tags, it is now possible to combine peptide solutions from up to 16 samples together before LC-MS/MS analyses. The quantitative advantage of using multiplexing isobaric compounds is that these molecules contain a reporter ion that is cleaved off during high collision dissociation together with precursor ion fragmentation. Therefore, quantifications of the reporter ions are performed at the MS2 level in the same spectrum used for peptide identification. Nevertheless, state-of-the-art equipment capable of MS3 or quantifications based on complement reporter ions are required to avoid quantification distortion due to interfering co-isolated ions (McAlister et al., 2014; Sonnett et al., 2018).

### 5.2 Combined Approaches for Selective Structural Analysis of Protein Complexes

Cross-linking mass spectrometry (XL-MS) has emerged as a powerful technology for interactome and structural biology studies in mitochondria (Schweppe et al., 2017; Liu et al., 2018; Ryl et al., 2020; Hevler et al., 2021b). Chemical cross-linkers react covalently with residues of proteins that are in close proximity to stabilize native PPIs for further characterization (Gomes and Gozzo, 2010; Mintseris and Gygi, 2020). After scrutiny of the identified intra- or inter-cross-linked peptides, a list of protein interactors and distance restraints is generated. This information can be used to guide computational modeling of single proteins and complex interfaces, most often in combination with data derived by classical structural methods, such as X-ray crystallography, nuclear magnetic resonance (NMR) or cryogenic electron microscopy (cryo-EM) (Herzog et al., 2012; Kornberg et al., 2015; O'Reilly and Rappsilber, 2018; Steigenberger et al., 2020).

Before structure analysis, determination of the optimal concentrations of cross-linkers and protein is strictly required to abate artifacts. To help circumvent this, a hybrid workflow termed in-gel cross-linking (IGX-MS) has recently been developed (Hevler et al., 2021a). In this method, proteins are separated by BN-PAGE and the gel spots of interest are cut and incubated with a cross-linker, digested and further analyzed by LC-MS/MS.

IGX-MS not only makes time-consuming experimental optimization steps (e.g., determining concentration of cross-linkers, optimal buffer system, etc.) nearly obsolete but also decreases the required protein amounts as well as undesired over-length cross-links. In contrast to classical in-solution XL-MS workflows, IGX-MS allows the differentiation of conformation- and interaction-specific distance restraints as it has been shown for various protein complexes, among them complexes I and V from bovine heart mitochondria. IGX-MS seems specially promising for future modeling studies that aim at characterizing co-occurring protein complexes with different stoichiometries or assembly states.

It has lately been demonstrated that CP and XL-MS are highly complementary and could provide valuable insights into the macromolecular organization of, for instance, the mitochondrial complexome. By combining CP and XL-MS data, Hevler and co-workers build a detailed atomic model of bovine CIV associated with dimeric apoptosis-inducing factor 1 (AIFM1) (Hevler et al., 2021b). CP analysis was indeed helpful to determine the stoichiometry of such a complex and validating this interaction. Combination of both approaches offers the possibility to cross-validate PPIs and to identify protein complexes that could be overlooked otherwise. Although actual scalability and throughput have not yet been addressed, combination of CP and XL-MS holds a great potential not only to better define the composition and states of protein complexes but also to help stabilize transient PPIs.

XL-MS has also been used to validate another CP-like setup that includes protein separation by SEC, quantitative MS analysis, cryo-electron microscopy (EM) and computational modeling. This structural proteomics approach allowed simultaneous description of the abundance, PPIs and structure profiling of 1/3 of the proteome of *Chaetomium thermophilum* (Kastritis et al., 2017). These results were integrated in a network map comprising 48 protein complexes and communities. This approach was also suitable to resolve the structure of the fatty acid synthase complex and its arrangements. Recent inclusion of image-processing workflows based on machine-learning methods opens the door to a much more robust data analysis, improved identification of PPIs, higher resolution in structure models and multi-scale molecular description of protein communities *in situ* (Kyrilis et al., 2021).

### 5.3 Investigating DNA-/RNA-Protein Complexes by Complexome Profiling

CP has not been popular for the analysis of DNA-interacting protein complexes. These have been addressed with more targeted approaches like chromatin immunoprecipitation (ChIP) (Das et al., 2004), electrophoretic mobility shift assay (EMSA) (Hellman and Fried, 2007) and a variety of Co-IP techniques (Sahr and Buchrieser, 2013), which have been extensively reviewed by Ferraz et al. (2021). Although both genomic and mitochondrial DNA molecules that interact with proteins are too large to enter standard native gels and do not exist in populations separable by size, like RNPs, CP could be useful for decomposing DNA-associated protein complexes. For example, Munawar and co-workers studied the complexome of a chromatin-enriched fraction separated by BN-PAGE after enzymatic digestion of DNA (Munawar et al., 2015). The resulting profile contained >50% of known chromatin complexes represented by at least half their subunits and contained information about assemblies for some of the complexes, such as PRC2 and NuRD. This illustrated the potential of CP for massive characterization of DNA-associated complexes. This fact is also corroborated by appearance of a variety of DNA-related complexes in existing CP datasets, such as histones, DNA repairing, replication and transcription factors.

Two research groups aiming to characterize the protein composition of chromatin complexes have made use of separation of protein complexes by size and LC-MS/MS analysis followed by correlation profiling. One utilized sucrose density gradients for separation of complexes containing chromatin-associated protein Wdr82 (Lee and Skalnik, 2013). The other used BN-PAGE to separate different populations of a chromatin remodeling complex NuRD derived from mouse embryonic stem cells. This approach led, among other things, to discovery of a new subunit of NuRD-associated protein, Wdr5 (Bode et al., 2016). Although these works were performed using affinity-purified complexes, they illustrated the potential of CP for characterization of chromatin-associated protein complexes as both allowed identification of different populations and assemblies in native state in contrast to pull-down techniques.

Conversely, CP variants to study RNA-protein complexes have been developed. Yet, these approaches have been particularly used for studying ribonucleoproteins (RNPs). Applications of RNP complexomics are generously reviewed in Gerovac et al. (2021). Complexomics studies based on protein co-migration profiles obtained from density gradients are commonly used to investigate ribosomal protein components and associated factors (Yu et al., 2005; Aviner et al., 2017; Van Haute et al., 2019; Palenikova et al., 2021a). Besides ribosomes, other RNPs are likely to have specific sedimentation patterns on density gradients due to their complex composition (Caudron-Herger et al., 2019). The simplicity and gentleness of fractionation by density gradient allows parallel identification of both proteins and RNA molecules by LC-MS/MS and RNA-seq, respectively. This setup has been established in a robust RNP complexomics method called Grad-seq that enabled global classification of stable RNA-protein complexes as well as identification of novel RNPs (Smirnov et al., 2016; Hor et al., 2020).

SEC is another method that has successfully been used for separation of RNPs from complex mixtures (Yoshikawa et al., 2018; Mallam et al., 2019). It provides fast and reproducible automated separation of particles with an impressive upper limit size of ∼30–40 MDa. SEC has been applied to specifically separate human 80S monosomes (∼4.3 MDa) and n-polysomes from HeLa cells (Yoshikawa et al., 2018). Moreover, hierarchical clustering revealed complexes involved in translation associated with ribosomal subunits, which makes this method a good alternative to the time-consuming and less specific DGU.

Classic CP has also been useful for dissecting protein composition of mitoribosomes as they enter regular BN-gels (Wessels et al., 2013; Chatzispyrou et al., 2017; Gardeitchik et al., 2018; Van Strien et al., 2019; Cunatova et al., 2021). For larger ribosomal species however, LP-native gels can be used (Strecker et al., 2010). As aforementioned, LP-BN-PAGE-based CP helped identify a RNP complex containing the mt-SSU associated with several translation and transcription factors at ∼5.7 MDa (Rugen et al., 2019). LP-gels could thus be very useful for further dissection of larger RNPs or those that are associated with many other factors as it has been commonly reported for translation and RNA-processing machineries (Acestor et al., 2009; Hocine et al., 2010; Blombach et al., 2011; Doetsch et al., 2011; Sprink et al., 2016; Duss et al., 2019; Gopalakrishna et al., 2019; Hilander et al., 2021).

A potential issue for characterization of RNA-protein complexes may result from the poor stability of RNA molecules, which are also prone to degradation by RNases. In this case, RNA-protein interactions would be lost and leading to identification of not only sub-assemblies but also artifacts. To avoid this, prior to CP, separation of these complexes should be thus performed using RNase-free materials and RNase inhibitors to keep stable both the protein and RNA interactors (López De Heredia and Jansen, 2004; Mili and Steitz, 2004; Wang et al., 2016; Aarum et al., 2020). Alternatively, to stabilize weak interactions of fragile RNA-protein complexes during separation, cross-linking strategies can be implemented. Treatment with chemicals (Rugen et al., 2019; Herrmannova et al., 2020; Patton et al., 2020), such as formaldehyde or UV irradiation (Trendel et al., 2019; Urdaneta et al., 2019; Urdaneta and Beckmann, 2020; van Esveld and Spelbrink, 2021) create short cross-links between a nucleic acid and close-contact protein interactors. In contrast, different types of enzymatic digestion of RNA can be used purposely to affect structure and stability of RNA-protein complexes to build additional levels of information for CP analysis, which can enable identification of previously unknown RNA-binding proteins (Caudron-Herger et al., 2019; Mallam et al., 2019; Gerovac et al., 2021).

### 5.4 Filling the Void: Complexome Profiling as a Workflow to Identify Protein-Protein Interactions and Improve Functional Annotation of Protein Complexes Across Species

Many species still lack functional annotation for a large portion of their genomes despite their significance as pathogens or drivers of major ecological processes. This is often owed to poor conservation, missing orthologues and the additional pitfall that the most unique biology that is not found in standard models, remains enigmatic. Systematic knowledge of PPIs can allow us to place these proteins into pathways, compartments and interaction networks. CP represents a clear choice to help narrow this gap and has the added benefit that no genetic intervention is required, which allows immediate assessment of species without a well-developed toolkit.

For example, Hillier et al. applied CP to study schizont stages of three different *Plasmodium* species. Integration with machine-learning led to identification of ∼20,000 putative PPIs arranged into ∼600 protein clusters, which were used to map the interactome (Hillier et al., 2019). Next to creating a valuable resource for *Plasmodium* research, the authors shed light on the interaction network of a group of parasite-specific transcription factors as well as identifying novel parasite-specific interactors. The latter could serve as targets for drug development due to their divergence from host biology. These data are sorely needed in the face of emerging drug resistance that is threatening our attempts to eradicate malaria (Haldar et al., 2018).

Prokaryotes also deviate considerably from the standard protein complexes found in eukaryotes. These organisms cover virtually every ecological niche on earth and have adapted their protein complexes accordingly. This is indeed exemplified in energy metabolic pathways, where prokaryotes utilize a wide variety of electron donors and acceptors, each requiring their own adapted set of classical and alternative respiratory complexes (Poole and Cook, 2000; Kaila and Wikstrom, 2021), most of which remain undescribed. While genomic analyses led to the hypothetical annotation of many homologous proteins involved in prokaryotic respiration, this approach is ill suited to the identification of novel components and interactors. CP complements this shortcoming and stands up as a good approach for characterizing PPIs in these organisms. This was illustrated beautifully by de Almeida and co-workers who investigated the electron transport system of the anaerobic ammonium-oxidizing bacterium *Kuenenia stuttgartiensis* (de Almeida et al., 2016). These bacteria oxidize ammonium with nitrite to produce N_2_ gas. It has been estimated that this process account for ∼50% of all N_2_ emitted into the atmosphere (Devol, 2015). Although this enormous ecological significance, the underlying respiratory complexes were largely theoretical (Kartal et al., 2013). By applying CP, these authors did not only confirm the presence of nearly all predicted respiratory complexes but also found evidence for novel protein complexes. One striking finding was that this bacterium appears to assemble a set of respiratory complexes that utilize Na^+^ besides the expected proton-pumping complexes, which suggested a role for a sodium-motive force to drive, for instance, the unfavorable reduction of ferredoxin. A set of three Rieske/cyt*b* complexes has also been found that shed light on how these bacteria can capture energy from hydrazine oxidation, challenging a previous hypothesis suggesting a quinone-reducing enzyme (Kartal et al., 2013). Similarly, CP has been used to analyze the nitrite-dependent methanotroph *Methylomirabilis lanthanidiphila* (Versantvoort et al., 2019). This species may potentially contribute to reduction of methane and nitrous oxide; i.e., potent greenhouse gases, by oxidizing methane to CO_2_ and reducing nitrate without producing nitrous oxide. To explain this process, a metabolic model based on genomic information had previously been proposed (Ettwig et al., 2010). Versantvoort and co-workers could confirm this model by identifying all proposed underlying protein complexes. Interestingly, three protein complexes thought to be involved in nitrate reduction were also identified, suggesting metabolic capabilities which had not yet been described in *Methylomirabilis* species. On the other hand, application of CP to study the marine sulfate reducer *Desulfobacula toluolica*, also led to detection of not only the expected membrane complexes but also new assemblies, unexpected associations, multimeric states, redox complexes involved in Na^+^-based bioenergetics and indications of respiratory supercomplexes (Wöhlbrand et al., 2016).

To shed more light on the biogenesis of thylakoid membrane complexes, CP was used to study auxiliary factors involved in the assembly of photosystem II (PSII) from the single-cell green alga *Chlamydomonas reinhardtii* (Spaniol et al., 2021). Authors demonstrated that the homolog for low PSII accumulation 2 (LPA2) protein is critical for PSII assembly and further supercomplex formation. Besides, novel interactors were found and possibly involved in regulation of PSII. This study exemplifies the great potential CP has for further studying photosynthesis-related protein complexes in algae, cyanobacteria and plants.

Finally, even in microorganisms that are not currently obtainable in axenic cultures, such as the archaeon *Candidatus Methanoperedens*, CP has proven invaluable (Berger et al., 2021). In this study, Berger et al. confirmed the presence of several predicted membrane-bound respiratory chain complexes as well as novel associations. Taken together these results demonstrated the suitability of CP to explore species previously inaccessible to most protein research. Overall, it is clear that CP complements (meta)genomic studies perfectly, by identifying in one experiment how these gene products assemble to form protein complexes, what subset of genome is really expressed and consequently update our understanding of metabolic capabilities and how these organisms achieve them.

### 5.5 Exploring Effects of Pathogenic Mutations in Non-Mitochondrial Proteins by Complexome Profiling

Although to date the vast majority of CP analyses in patients have been focused on disorders affecting mitochondria, its application is broadening nonetheless. The effects of pathogenic missense mutations in structural subunits of the V-type ATPase on the stability of the holoenzyme have been characterized by this method (Van Damme et al., 2017). By whole exome sequencing, predicted pathogenic mutations have been identified in genes *ATP6V1E1* and *ATP6V1A*, encoding subunits E and A from the V_1_ segment of the V-type ATPase, respectively. Primary skin fibroblasts from three patients, one with a mutation in E1 and two with mutations in subunit A were compared to controls and to an earlier described subject presenting cutis laxa with a mutation in subunit ATP6V0A2 of the V_O_ segment. To further investigate this, enriched Golgi preparations have been analyzed by BN-PAGE-based CP. Migration patterns of healthy controls revealed three assembly intermediates of the V_1_ segment, the V_O_ segment and a highly abundant, fully assembled V_1_V_O_-ATPase complex. In contrast, in patient fibroblasts with mutations in subunits of the V_1_ segment, only the V_O_ intermediate and traces of the holo-complex have been detected. Comparably, the mutation in ATP6V0A2 decreased the abundance of intermediates of both domains as well as the holo-complex. The destabilization of the fully assembled V_1_V_O_-ATPase evidenced by CP contributed to detailing the molecular consequences and pathogenicity of these mutations.

In a recent CP study of peroxisome-deficient cells using peroxins deletion strains of *S. cerevisiae* as a model system and fibroblasts from a Zellweger syndrome patient, Nübel and co-workers linked accumulation of miss-targeted peroxisomal membrane proteins into mitochondria to metabolic and morphological abnormalities of this organelle (Nuebel et al., 2021). Complexome profiles of yeast strains lacking peroxisomes showed formation of the docking subassembly of the peroxisomal importomer complex in mitochondria, explaining also the incorporation of peroxisomal matrix proteins to this subcellular compartment. It would be interesting to also explore the complexome profiles of fibroblasts from patients with Zellweger spectrum disorder to investigate in detail the rearrangements of mitochondrial complexes caused by peroxisomes deficit in humans.

These two studies exemplified how CP offers a straightforward option to shed light on the pathophysiological roles of critical proteins located in cell compartments other than mitochondria and the specific consequences of impairments in protein complexes and PPIs resulting from genetic and/or biochemical defects. CP thus holds a great potential to help not only unveil the molecular mechanisms of diseases, but also develop novel treatments.

## 6 Concluding Remarks

In a very short time, CP has revolutionized the way we study mitochondrial protein complexes and significantly increased the amount of data collected from single unbiased experiments. Research findings obtained by this methodology include, for instance, identification of novel OXPHOS-related subunits and assembly factors; stepwise description of intricate assembly pathways; molecular evidence on the role of accessory subunits and existence of tissue-specific subunit isoforms of CI; better insight on the formation of respirasomes and the supramolecular organization of the MICOS/MIB complexes as well as the unique composition of OXPHOS complexes and mitoribosomes in different species. Notably, CP has also been invaluable to better understand the effects of mutations found in mitochondrial genes from patients as well as in disease models. Recent developments in duplexing and multiplexing CP experiments not only enable accurate proteomic quantifications, but also greatly increase the feasibility of including this approach in future clinical diagnosis. We have also discussed innovative strategies exemplifying how CP easily complements other state-of-the-art methods such as XL-MS, RNAseq and cryo-EM. However, there is still a lot of room for improving this method and keep expanding its reach. It is projected that CP will further evolve with the advent of the next generation of LC-MS instruments and proteomic strategies as well as upcoming complexome data analysis software. Finally, we also encourage the scientific community to apply CP for studying protein complexes from cell compartments other than the mitochondrion and share publicly the resultant complexome datasets through CEDAR.
